# Supplementary material for: Structural basis of diverse antibody recognition of conserved coronavirus spike S2 epitopes that contribute to protective immunity
Source: bioRxiv. 2026 Sep 16:2026.09.13.750764. Preprint. [Version 1] doi: 10.64898/2026.09.13.750764 (PMC13596270; doi:10.64898/2026.09.13.750764)
Supplement: Supplementary file 1 [file NIHPP2026.09.13.750764v1-supplement-1.pdf]

bioRxiv preprint doi: https://doi.org/10.64898/2026.09.13.750764; this version posted September 16, 2026. The copyright holder for this preprint (which was not certified by peer review) is the author/funder, who has granted bioRxiv a license to display the preprint in perpetuity. It is made available under a CC-BY 4.0 International license.

Supplementary materials

Structural basis of diverse antibody recognition of conserved coronavirus spike S2 epitopes that contribute to protective immunity

Adonis A. Rubio, Virginia Crivelli, Václav Hönig, Monika Čížková, Tomás Cervantes Rincón, Morgan E. Abernathy, Megan K. Parada, Danielle Vahdat, Yu E. Lee, Michael Eso, Jasmine Cantergiani, Simone Moro, David Jarrossay, Concetta Guerra, Benedetta Cena, Mihai Minculescu, Vaiva Gradauskaite, Maira Biggiogero, Veronica Calvaruso, Alessandra F. Pellanda, Andrea Cavalli, Christian Garzoni, Martin Palus, Davide F. Robbiani, and Christopher O. Barnes

The PDF file includes:

Table S1. Amino acid sequence of coldspots and corresponding peptides. Related to Fig. 1.

Table S2. Binding kinetics of CH and LS monoclonal antibodies. Related to Fig. 2.

Table S3. X-ray crystallography data collection and refinement statistics. Related to Fig. 2 and 3.

Table S4. Cryo-EM data collection and refinement statistics. Related to Fig. 3 and 4.

Figure S1. Identification of coldspot antibodies to the SARS-CoV-2 spike internal fusion peptide, central helix, connector domain, and lower stalk, related to Fig. 1.

Figure S2. Profiling of coldspot monoclonal antibodies' binding and neutralization of SARS-CoV-2 pseudovirus, related to Fig. 1.

Figure S3. Cryo-EM data processing and validation of ch.005, ch.007, and ch.010 Fabs bound to SARS-CoV-2 stabilized stem (SS), related to Fig. 3.

Figure S4. Cryo-EM data processing, validation, and characterization of ch.008, related to Fig. 3.

Figure S5. Characterization of epitope and paratope contacts for ch.005, ch.007, and ch.010, related to Fig. 3 and 4.

Figure S6. *In vitro* Fc-mediated effector functions by CH and LS antibodies, related to Fig. 5.

Figure S7. LS-directed antibody ls.019 does not confer protection *in vivo*, related to Fig. 5.

Other supplementary material for the manuscript includes the following:

Data File S1. Sequences of anti-coldspot antibodies.

Data File S2. Sequences and neutralizing and binding data of the monoclonal antibodies.

Data File S3. Alignment of the SARS-CoV-2 coldspot peptide sequences to related CoV species.

Table S1. Amino acid sequence of coldspots and corresponding peptides. Related to Figure 1.

Coldspot Identifier	Bianchini, F. et al. 2023		Sequence of peptides used in this paper[c,d]	Evolution of coldspot sequence based on analysis with all sequences until July 2025	
	Residues[a]	Amino acid sequence[b,d]		Residues[a]	Amino acid sequence[d,e]
cs1 (Fusion Peptide)	814-838	KRSFIEDLLFNKVTLADAGFIKQYG	PSKRSFIEDLLFNKVTLADA	813-844	(S)KRSFIEDLLFNKVTLADAGFIKQYG(DCLGDI)
cs2 (Stem Helix)	1142-1161	QPELDSFKEELDKYFKNHTS	ELDSFKEELDKYFKNHTSPD	1144-1161	[~~QP~~]ELDSFKEELDKYFKNHTS
cs3	113-137	KTQSLLIVNNATNVVIKVCEFQFCN	KTQSLLIVNNATNVVIKVCEFQFCN	113-126	KTQSLLIVNNATNV[~~VIKVCEFQFCN~~]
cs4	191-209	EFVFKNIDGYFKIYSKHTP	EFVFKNIDGYFKIYSKHTP	191-209	EFVFKNIDGYFKIYSKHTP
cs5	309-338	EKGIYQTSNFRVQPTESIVRFPNITNLCPF	EKGIYQTSNFRVQPTESIVRFPNITNLCPF	300-331	(KCTLKSFTV)EKGIYQTSNFRVQPTESIVRFPN[~~ITNLCPF~~]
cs6	377-404	FKCYGVSPTKLNDLCFTNVYADSFVIRG	FKCYGVSPTKLNDLCFTNVYADSFVIRG	377-402	FKCYGVSPTKLNDLCFTNVYADSFVI[~~RG~~]
cs7	418-438	IADYNYKLPDDFTGCVIAWNS	IADYNYKLPDDFTGCVIAWNS	418-434	IADYNYKLPDDFTGCVI[~~AWNS~~]
cs8	454-476	RLFRKSNLKPFERDISTEIYQAG	RLFRKSNLKPFERDISTEIYQAG	461-474	[~~RLFRKSN~~] LKPFERDISTEIYQ [~~AG~~]
cs9	523-546	TVCGPKKSTNLVKNKCVNFNFNGL	TVCGPKKSTNLVKNKCVNFNFNGL	523-546	TVCGPKKSTNLVKNKCVNFNFNGL
cs10	584-612	ILDITPCSFGGVSVITPGTNTSNQVAVLY	ILDITPCSFGGVSVITPGTNTSNQVAVLY	584-612	ILDITPCSFGGVSVITPGTNTSNQVAVLY
cs11	733-763	KTSVDCTMYICGDSTECSNLLLQYGSFCTQL	KTSVDCTMYICGDSTECSNLLLQYGSFCTQL	733-763	KTSVDCTMYICGDSTECSNLLLQYGSFCTQL
cs12 (Internal FP')	860-878	VLPPLLTDEMIAQYTSALL	VLPPLLTDEMIAQYTSALL	860-878	VLPPLLTDEMIAQYTSALL
cs13 (Internal FP'')	889-928	GAGAALQIPFAMQMAYRFNGIGVTQNVLYENQKLIANQFN	GAGAALQIPFAMQMAYRFNGIGV	884-935	(SGWTF)GAGAALQIPFAMQMAYRFNGIGVTQNVLYENQKLIANQFN(SAIGKIQ)
cs14 (Central Helix)	983-1019	RLDKVEAEVQIDRLITGRLQSLQTYVTQQLIRAAEIR	RLDKVEAEVQIDRLITGRLQ (cs14.1)<br>RLQSLQTYVTQQLIRAAEIR (cs14.2)	983-1019	RLDKVEAEVQIDRLITGRLQSLQTYVTQQLIRAAEIR
cs15 (Connector Domain)	1028-1070	KMSECVLGQSKRVDFCGKGYHLMSFPQSAPHGVVFLHVTYVPA	KMSECVLGQSKRVDFCGKGYHL (cs15.1)<br>MSFPQSAPHGVVFLHVTYVPAQE (cs15.2)	1028-1072	KMSECVLGQSKRVDFCGKGYHLMSFPQSAPHGVVFLHVTYVPA(QE)
cs16 (Lower Stalk)	n/a	*not identified as a coldspot (<18 aa)*	VNIQKEIDRLNEVA	1177-1190	VNIQKEIDRLNEVA

[a]Numbering based on SARS-CoV-2 UniProt (P0DTC2) entry.

[b]Blue letters indicate residues present in the coldspot sequences identified in Bianchini et al. 2023 but absent in the peptide used in this paper.

[c]Green letters indicate residues present in the peptide sequence used in this paper but absent in the coldspot sequences identified in Bianchini et al. 2023.

[d]Red or underlined letters highlight the correspondence of residues between peptides used in this paper and coldspot sequences identified in Bianchini et al. 2023 or in this paper.

[e]Brackets and parentheses, [~~X~~] and ( ), indicate residues that were identified as part of the coldspot in Bianchini et al. 2023 but not in this paper, or that were identified as part of the coldspot in this paper but not in Bianchini et al. 2023, respectively.

bioRxiv preprint doi: https://doi.org/10.64898/2026.09.13.750764; this version posted September 16, 2026. The copyright holder for this preprint (which was not certified by peer review) is the author/funder, who has granted bioRxiv a license to display the preprint in perpetuity. It is made available under a CC-BY 4.0 International license.

Table S2. Binding kinetics of CH and LS monoclonal antibodies. Related to Figure 2.

Antibody	Antigen	k_{on} ($M^{-1}s^{-1}$)	k_{off} (s^{-1})	K_d (nM)
**ch.005**	SARS-CoV-2 SS	3.6×10^5	3.6×10^{-4}	1.0
	SARS-CoV-2 D994A SS	2.6×10^2	6.1×10^{-2}	2.3×10^6
	SARS-CoV-2 R995A SS	2.2×10^5	5×10^{-4}	2.3
	SARS-CoV-2 Q1002A SS	N.D.	N.D.	N.D.
**ch.007**	SARS-CoV-2 SS	1.4×10^5	1.5×10^{-4}	1.1
	SARS-CoV-2 D994A SS	4.7×10^3	1.5×10^{-3}	317
	SARS-CoV-2 R995A SS	N.D.	N.D.	N.D.
	SARS-CoV-2 Q1002A SS	3.6×10^4	5.7×10^{-4}	16
**ch.010**	SARS-CoV-2 SS	3.7×10^5	5.3×10^{-4}	1.4
	SARS-CoV-2 D994A SS	2.1×10^4	1.7×10^{-2}	802
	SARS-CoV-2 R995A SS	5.3×10^5	4.5×10^{-3}	8.4
	SARS-CoV-2 Q1002A SS	1.5×10^5	5.4×10^{-4}	3.4
	MERS-CoV SSv2	1.5×10^5	1.7×10^{-2}	115
**54043-5**[a]	MERS-CoV SSv2	2.1×10^5	2.2×10^{-3}	10
**ls.015**	SARS-CoV-2 spike 6P	2.2×10^4	3.2×10^{-3}	148
**ls.019**	SARS-CoV-2 spike 6P	1.1×10^4	1.3×10^{-3}	114

[a]Johnson, N.V. *et al.* 2024

[b]N.D., not determined

bioRxiv preprint doi: https://doi.org/10.64898/2026.09.13.750764; this version posted September 16, 2026. The copyright holder for this preprint (which was not certified by peer review) is the author/funder, who has granted bioRxiv a license to display the preprint in perpetuity. It is made available under a CC-BY 4.0 International license.

Table S3. X-ray crystallography data collection and refinement statistics. Related to Figure 2 and 3.

	ch.005 Fab	ls.019 scFv-LS peptide
**PDB ID**	**10QO**	**10NI**
**Data collection**[a]		
Space group	P 41	P 21 21 2
Unit cell (Å)	69.545, 69.545, 86.108	133.257, 38.765, 47.774
α, β, γ (°)	90, 90, 90	90, 90, 90
Wavelength (Å)	0.979	0.979
Resolution (Å)	36.61 – 1.6 (1.63 – 1.6)	38.83 – 1.788 (1.81 – 1.79)
Unique Reflections	53,716	23,663
Completeness (%)	99.4 (92.9)	99.2 (94.0)
Redundancy	7.3 (4.0)	4.4 (4.2)
$CC_{1/2}$ (%)	0.998 (0.794)	0.992 (0.352)
I / σI	14.8 (2.6)	5.2 (0.6)
Mosaicity (˚)		0.24
R_{merge} (%)	0.066 (0.612)	0.109 (1.56)
R_{pim} (%)	0.038 (0.467)	0.080 (1.14)
Wilson *B*-factor	18.76	26.37
**Refinement and Validation**		
Resolution (Å)	1.6	1.79
Number of atoms		
Protein	6,031	1,741
Ligand	0	0
Waters	34	57
R_{work}/R_{free} (%)	0.2112 / 0.2279	0.2159 / 0.2473
R.m.s. deviations		
Bond lengths (Å)	0.005	0.002
Bond angles (˚)	0.799	0.61
MolProbity score	0.89	1.62
Clashscore (all atom)	0.5	2.36
Poor rotamers (%)	1.17	2.75
Ramachandran plot		
Favored (%)	97.09	96.05
Allowed (%)	2.91	3.51
Disallowed (%)	0.00	0.44
Average *B*-factor (Å)	26.67	36.3

[a]Numbers in parentheses correspond to the highest resolution shell

Table S4. Cryo-EM data collection and refinement statistics. Related to Figure 3 and 4.

	**ch.005 SARS-CoV-2 SS (global)**	**(local)**	**ch.007 SARS-CoV-2 SS (global)**	**(local)**	**ch.008 SARS-CoV-2 SS (global)**	**(local)**	**ch.010 SARS-CoV-2 SS (global)**	**(local)**
**PDB**	**9ZLQ**	**9ZLR**	**9ZLS**	**9ZLT**	n/a	n/a	**n/a**	**9ZLU**
**EMD**	**-74407**	**-74408**	**-74409**	**-74410**	n/a	n/a	**-74411**	**-74412**
**Data collection conditions**								
Microscope	Krios G2		Krios G2		Krios G2		Krios G2	
Camera	K3		Falcon 4i		Falcon 4i		Falcon 4i	
Magnification	105,000x		130,000x		130,000x		130,000x	
Voltage (kV)	300		300		300		300	
Dose rate (e^-/pixel/s)	14.6		12.6		12.3		9.43	
Electron dose ($e^-/Å^2$)	50		50		50		50	
Defocus range (µm)	-1 to -2.2		-0.9 to -2.3		-1 to -2.5		-1 to -2.3	
Pixel size (Å)	0.85		0.92		0.92		0.92	
Micrographs collected	10,819		12,711		10,020		8,893	
Micrographs used	8,218		10,722		5,016		8,031	
Total extracted particles	2,960,049		2,606,196		295,145		1,655,752	
Final refined particles	189,046	567,138	1,040,243	3,120,729	62,407	187,221	259,207	777,621
Symmetry imposed	C3	C1	C3	C1	C3	C1	C3	C1
Nominal Map Resolution (Å)								
FSC 0.143 (unmasked/masked)	3.7/3.3	3.8/3.2	3.1/2.7	3.1/2.7	3.7/3.2	4.1/3.4	3.5/3	3.6/3
**Refinement and Validation**								
Initial model	8VCR		8VCR, 7LS9		n/a		8VCR, 7CDJ, 6URM	
Model Resolution (Å)								
FSC 0.143	3.2	3.1	2.6	2.6	n/a		n/a	3
Number of atoms								
Protein	15,110	3,825	14,449	4,416	n/a		n/a	3,395
Ligand	30	2	27	3	n/a		n/a	1
MapCC (global/local)					n/a		n/a	
Map sharpening B-factor	-115	-97.5	-119.4	-73.6	n/a		n/a	-65.8
R.m.s. deviations								
Bond lengths (Å)	0.003	0.003	0.002	0.002	n/a		n/a	0.003
Bond angles (°)	0.599	0.537	0.52	0.512	n/a		n/a	0.869
MolProbity score	1.74	1.71	1.78	1.73	n/a		n/a	1.56
Clashscore (all atom)	6.18	8.33	8.26	8.15	n/a		n/a	5.2
Poor rotamers (%)	0.74	0	0.27	0.21	n/a		n/a	0.27
Ramachandran plot								
Favored (%)	94.01	96.14	95.18	95.82	n/a		n/a	95.86
Allowed (%)	5.99	3.86	4.82	4.18	n/a		n/a	4.14
Disallowed (%)	0	0	0	0	n/a		n/a	0

bioRxiv preprint doi: https://doi.org/10.64898/2026.09.13.750764; this version posted September 16, 2026. The copyright holder for this preprint (which was not certified by peer review) is the author/funder, who has granted bioRxiv a license to display the preprint in perpetuity. It is made available under a CC-BY 4.0 International license.

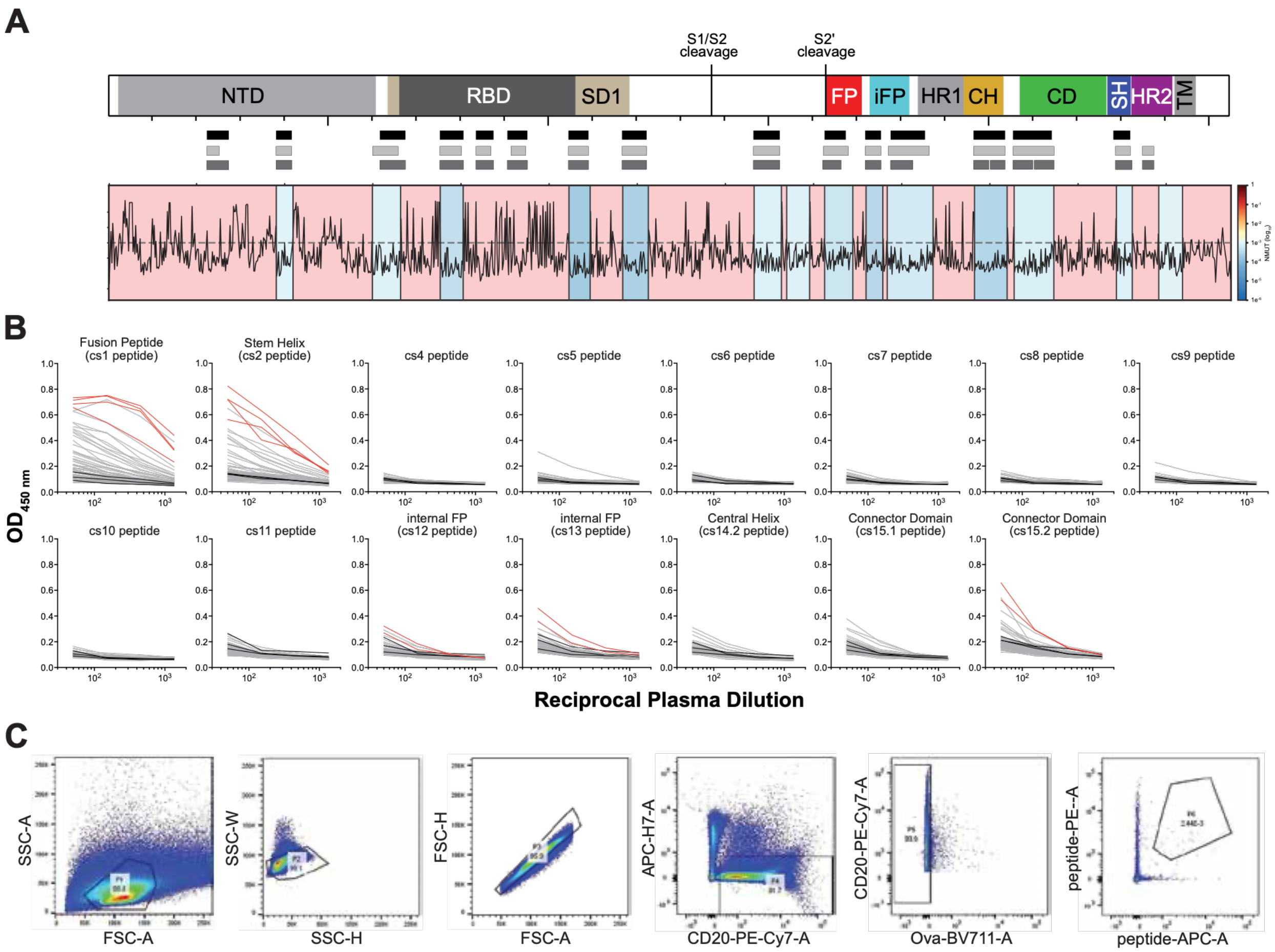

Figure S1. Identification of coldspot antibodies to the SARS-CoV-2 spike internal fusion peptide, central helix, connector domain, and lower stalk, related to Figure 1.

(A) Top: Cartoon diagram of the functional domains of the SARS-CoV-2 spike protein. Coldspot regions and corresponding peptides are shown with rectangles below the diagram as follows: top row, coldspots as identified in Bianchini et al (black); middle row, evolution of the coldspots from Bianchini et al. by taking into consideration of sequences until July 2025 (light grey); bottom row, coldspot peptides used in this paper (dark grey). See also Table S1. Bottom: mapping of the frequency of amino acid changes (until July 2025). **(B)** ELISA plots for plasma IgG reactivity to coldspot peptides in spike. Each line represents a convalescent individual; red lines indicate donors selected for antibody isolation; black lines indicate prepandemic controls. Note: a peptide corresponding to cs3 could not be synthesized. **(C)** Representative gating strategy for sorting S2 coldspot peptide-specific B cells by flow cytometry.

bioRxiv preprint doi: https://doi.org/10.64898/2026.09.13.750764; this version posted September 16, 2026. The copyright holder for this preprint (which was not certified by peer review) is the author/funder, who has granted bioRxiv a license to display the preprint in perpetuity. It is made available under a CC-BY 4.0 International license.

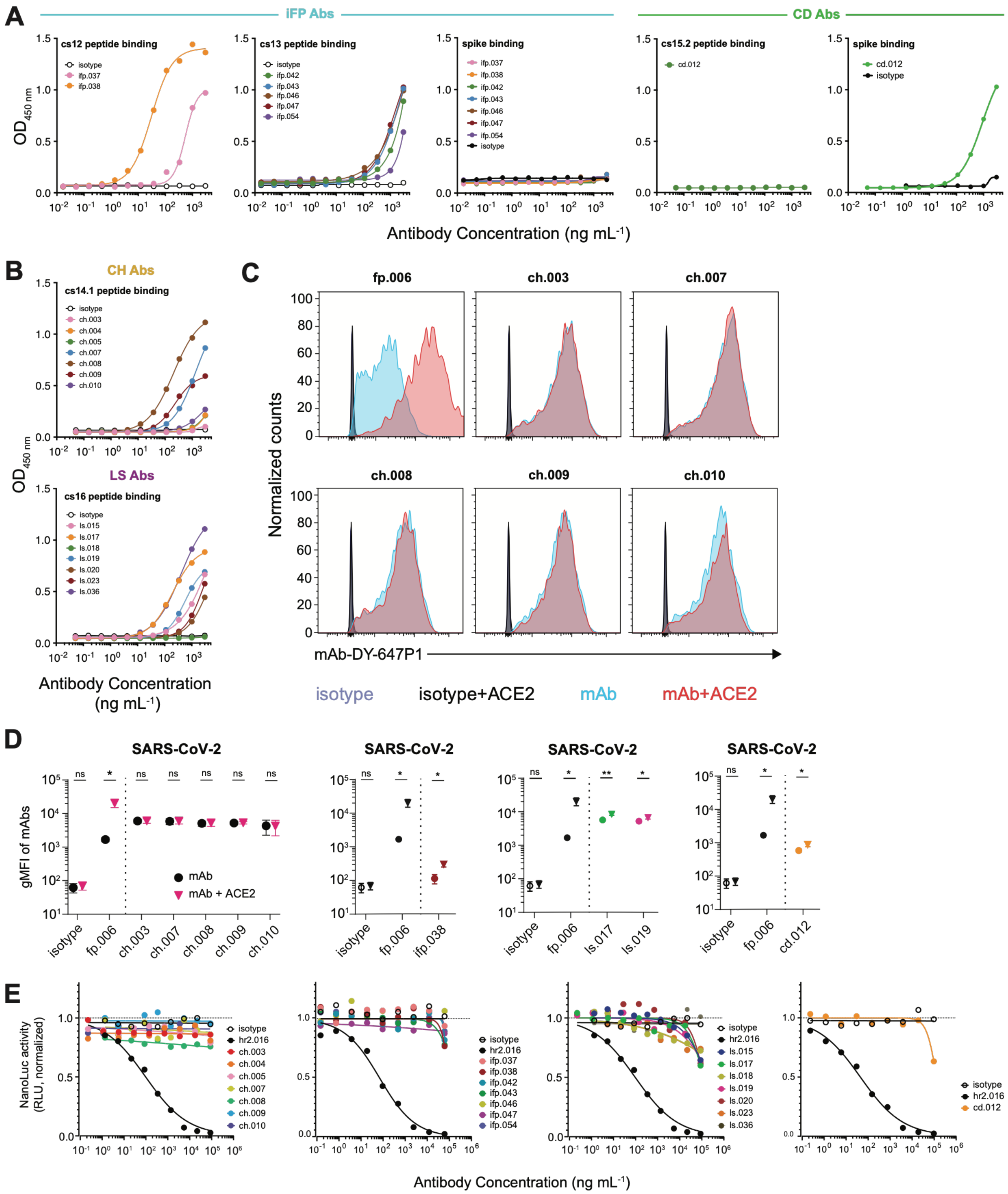

Figure S2. Profiling of coldspot monoclonal antibodies' binding and neutralization of SARS-CoV-2 pseudovirus, related to Figure 1.

(A-B) ELISAs measuring recombinant monoclonal antibody binding to (A) cognate peptide and SARS-CoV-2 spike for iFP (cs12 and cs13) and CD (cs15.2) and (B) CH (cs14.1) and LS (cs16) peptides. See Figure 1E for comparative binding of CH and LS antibodies to full-length spike. **(C)** Representative flow cytometry plots of anti-CH antibodies binding to SARS-CoV-2 spike expressed on HEK293 cells, in the presence or absence of soluble human ACE2. Isotype and anti-FP antibody fp.006 are shown as

bioRxiv preprint doi: https://doi.org/10.64898/2026.09.13.750764; this version posted September 16, 2026. The copyright holder for this preprint (which was not certified by peer review) is the author/funder, who has granted bioRxiv a license to display the preprint in perpetuity. It is made available under a CC-BY 4.0 International license.

references. **(D)** Quantification of the geometric mean fluorescence intensity (gMFI) for CH, iFP, CD, and LS antibodies against spike expressed on HEK293 cells, in the presence or absence of soluble human ACE2. **(E)** Represenative dose-response curves of antibody neutralizing capacity depicted as changes in normalized relative luminescence (RLU) in cell lysates 48h after infection with ancestral SARS-CoV-2 pseudovirus in the presence of increasing concentrations of CH, iFP, LS, and CD antibodies, from left to right, respectively.

bioRxiv preprint doi: https://doi.org/10.64898/2026.09.13.750764; this version posted September 16, 2026. The copyright holder for this preprint (which was not certified by peer review) is the author/funder, who has granted bioRxiv a license to display the preprint in perpetuity. It is made available under a CC-BY 4.0 International license.

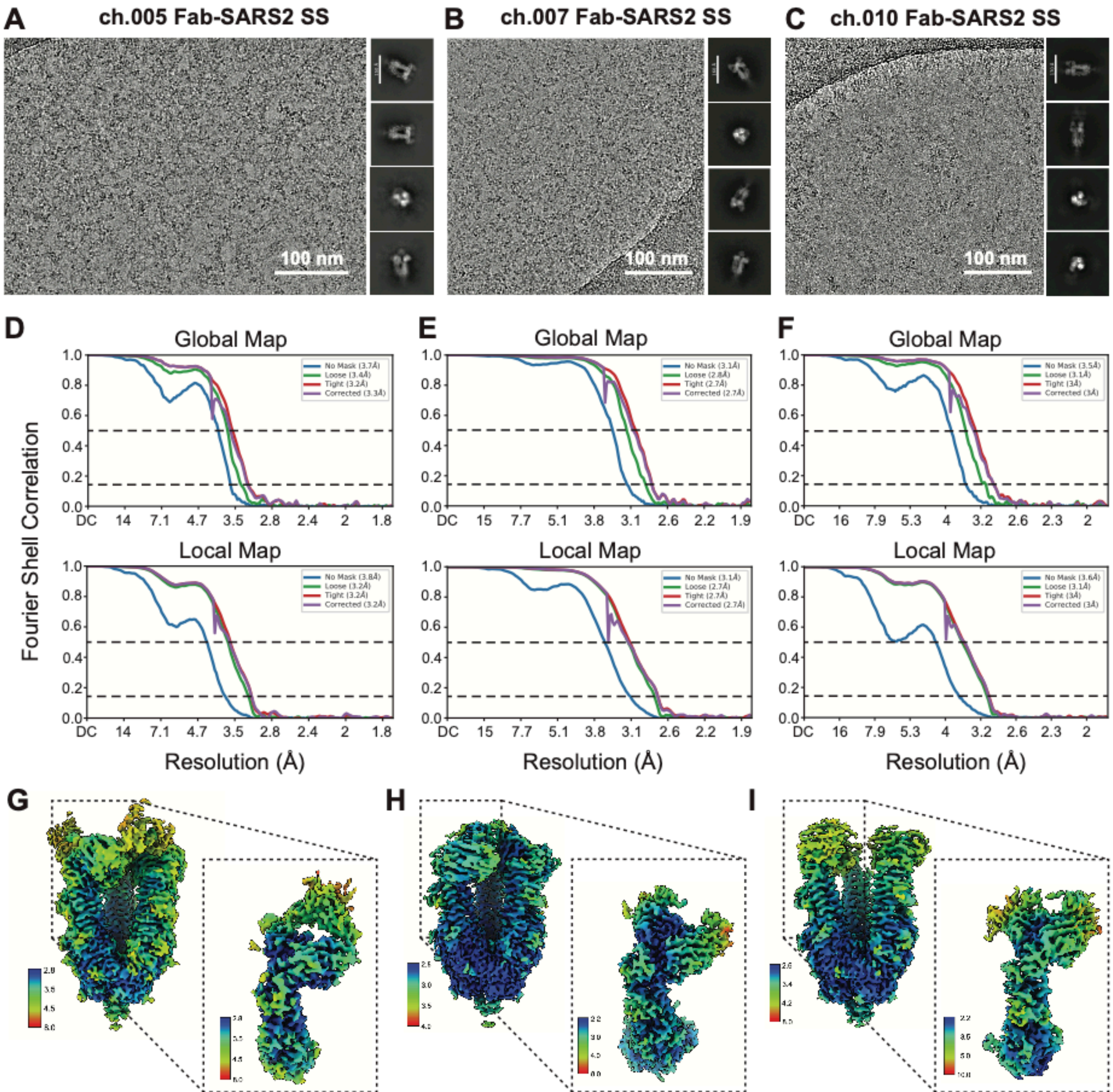

Figure S3. Cryo-EM data processing and validation of ch.005, ch.007, and ch.010 Fabs bound to SARS-CoV-2 stabilized stem (SS), related to Figure 3.

(A-C) Representative micrographs and 2D class averages for **(A)** ch.005, **(B)** ch.007, and **(C)** ch.010 Fabs complexed with SARS-CoV-2 SS (see Table S4). **(D-F)** Gold-standard FSC plots for the global (top) or local (bottom) refinements for **(D)** ch.005-SS, **(E)** ch.007-SS, and **(F)** ch.010-SS complexes. **(G-I)** Local resolution estimations for **(G)** ch.005-SS, **(H)** ch.007-SS, and **(I)** ch.010-SS. Insets depict local resolution estimations for the Fab-protomer focused refinements.

bioRxiv preprint doi: https://doi.org/10.64898/2026.09.13.750764; this version posted September 16, 2026. The copyright holder for this preprint (which was not certified by peer review) is the author/funder, who has granted bioRxiv a license to display the preprint in perpetuity. It is made available under a CC-BY 4.0 International license.

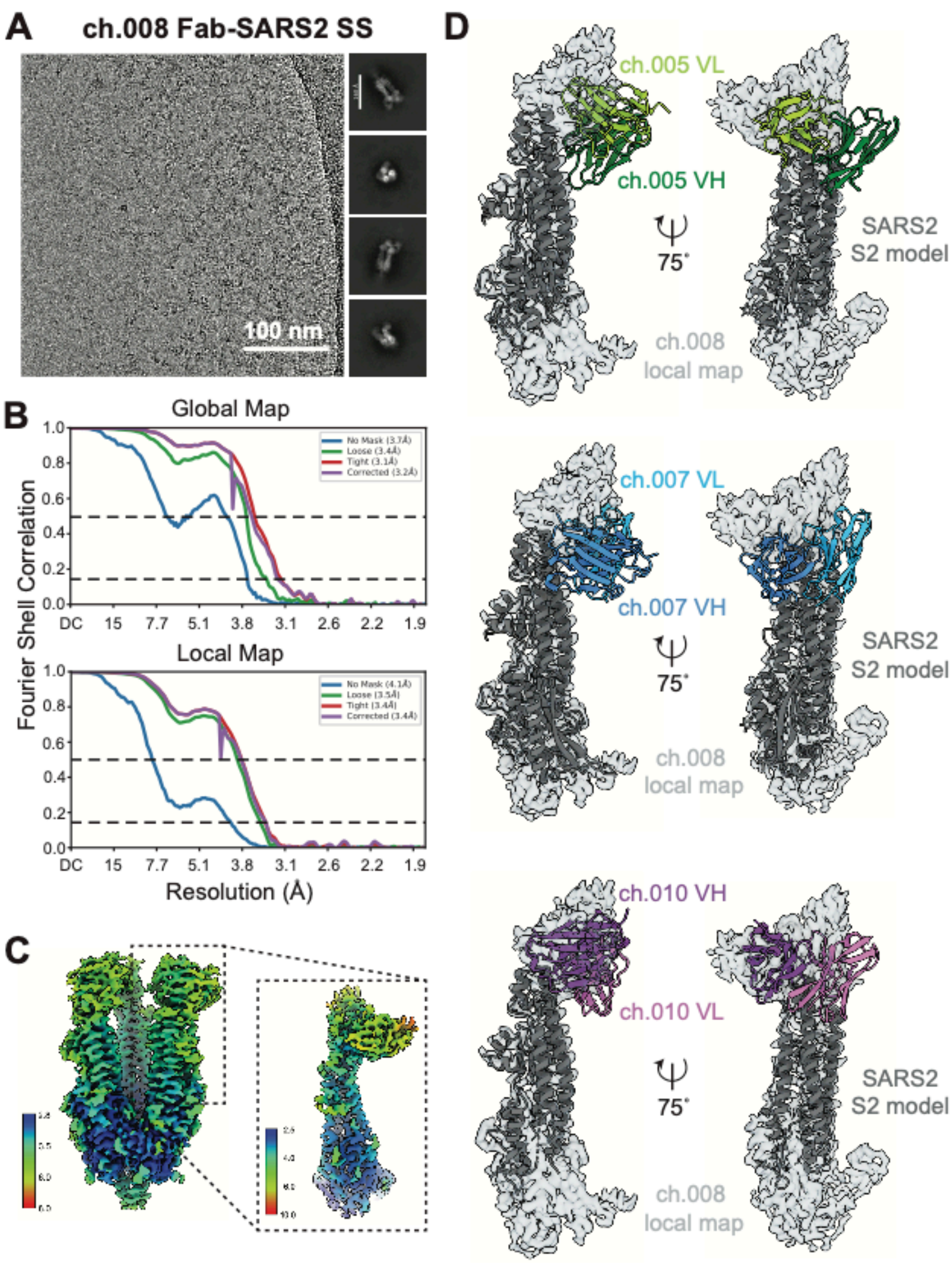

Figure S4. Cryo-EM data processing, validation, and characterization of ch.008, related to Figure 3.
(A) Representative micrographs and 2D class averages for ch.008 complexed with SARS-CoV-2 SS (see Table S4). **(B)** Gold-standard FSC plots for the global (top) or local (bottom) refinements for ch.008-SS. **(C)** Local resolution estimations for ch.008-SS. Insets depict local resolution estimations for the Fab-protomer focused refinements. **(D)** Dockings of local refinement models of ch.005-SS (top), ch.005-SS (middle), and ch.010-SS (bottom) into the ch.008-SS local refinement map for comparison of binding pose.

bioRxiv preprint doi: https://doi.org/10.64898/2026.09.13.750764; this version posted September 16, 2026. The copyright holder for this preprint (which was not certified by peer review) is the author/funder, who has granted bioRxiv a license to display the preprint in perpetuity. It is made available under a CC-BY 4.0 International license.

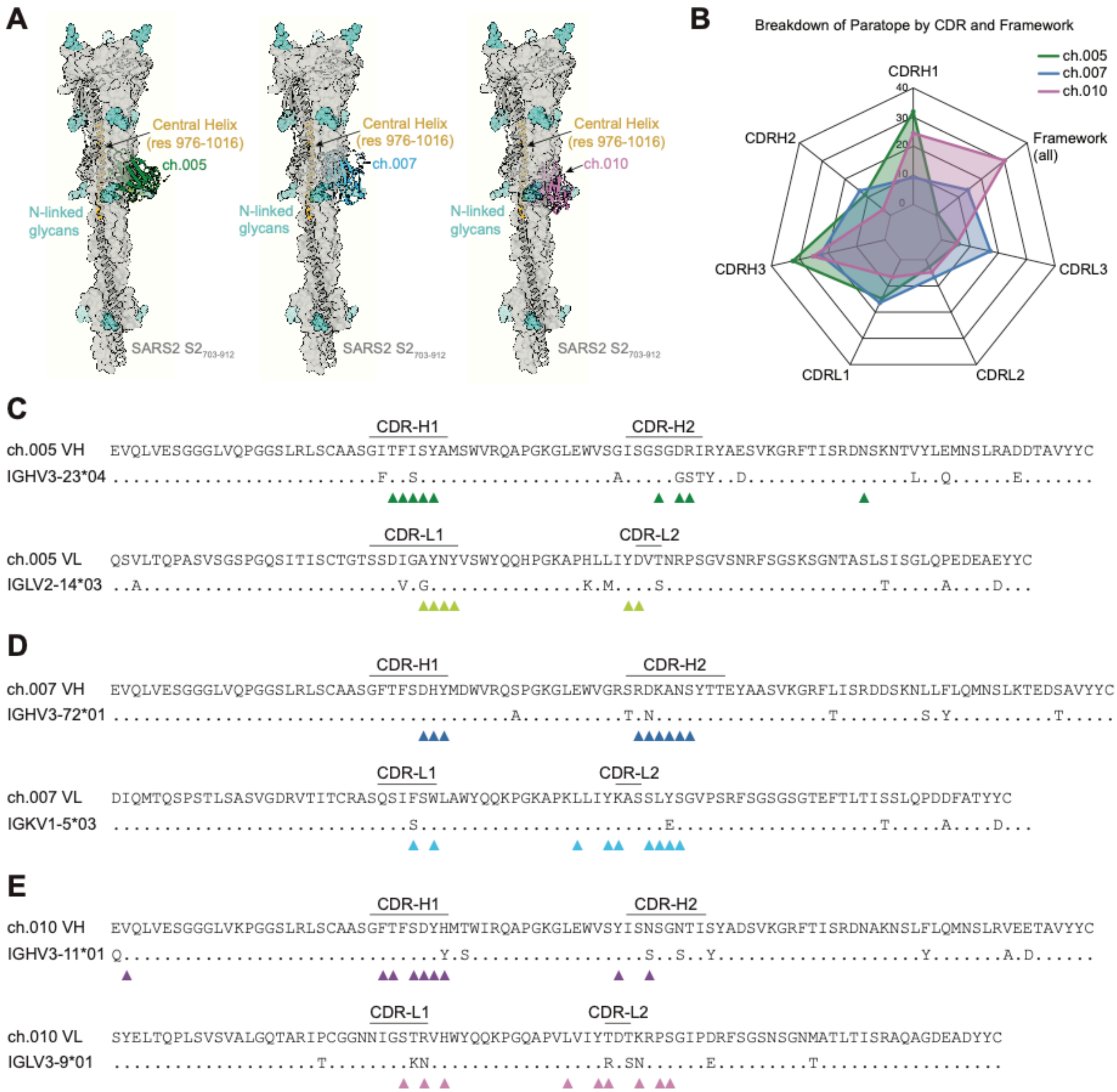

Figure S5. Characterization of epitope and paratope contacts for ch.005, ch.007, and ch.010, related to Figures 3 and 4. **(A)** Modeling of ch.005 (left), ch.007 (middle), and ch.010 (right) V_H-V_L engaging their epitopes in the SARS-CoV-2 postfusion state (PDB 7E9T). Models were generated by aligning Cα of residues 988-1028 of the local refinement models to the postfusion model. **(B)** Radar plot depicting the percent contribution of individual V_H and V_L CDR loops and total framework residues to the antibody paratopes for ch.005 (green), ch.007 (blue), and ch.010 (purple). **(C-E)** Sequence alignment of antibody V_H (top) or V_L (bottom) to their respective Ig germlines for ch.005 **(C)**, ch.007 **(D)**, and ch.010 **(E).** Dots represent agreement with germline. Residues involved with the paratope are denoted by triangles.

bioRxiv preprint doi: https://doi.org/10.64898/2026.09.13.750764; this version posted September 16, 2026. The copyright holder for this preprint (which was not certified by peer review) is the author/funder, who has granted bioRxiv a license to display the preprint in perpetuity. It is made available under a CC-BY 4.0 International license.

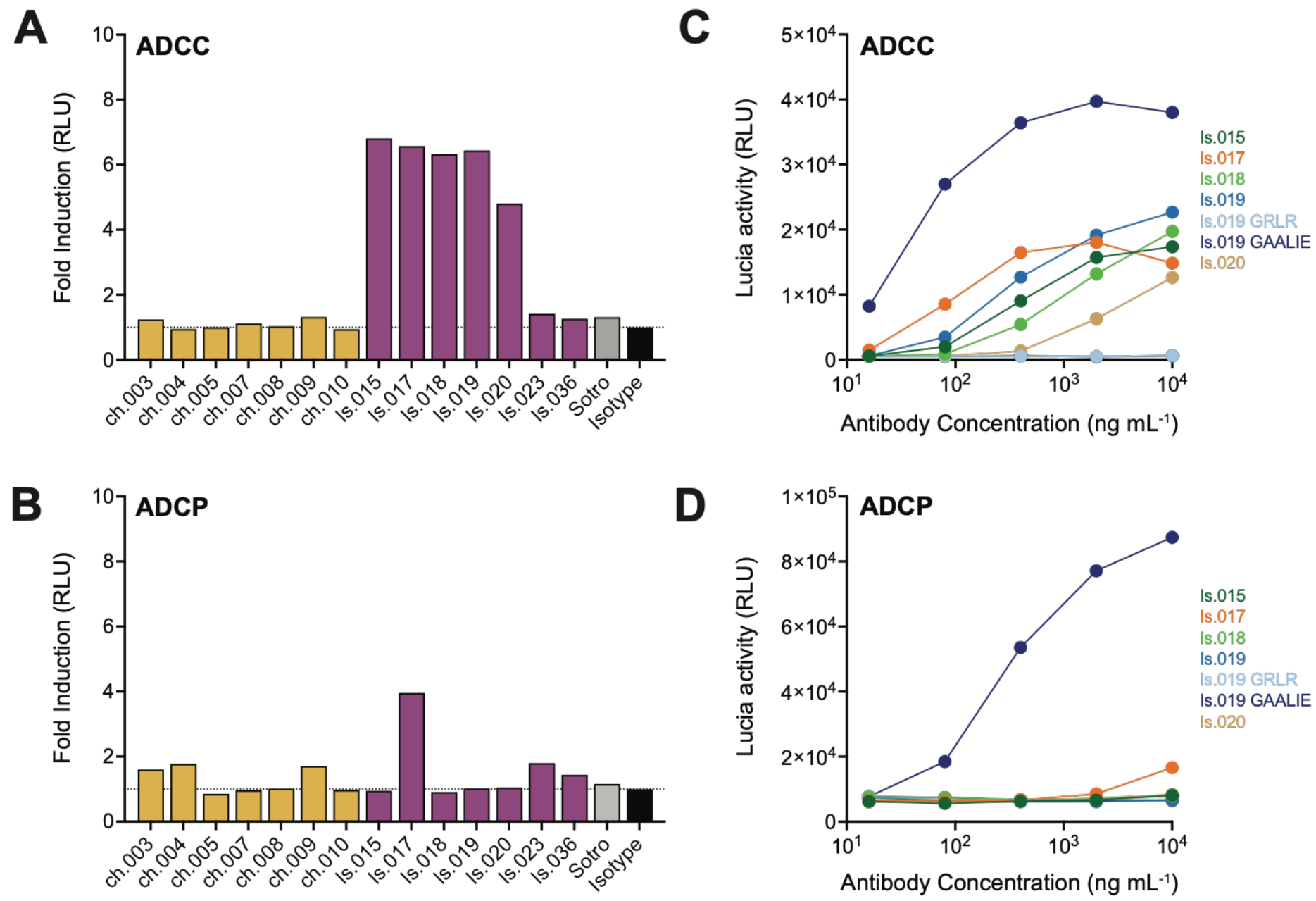

Figure S6. *In vitro* Fc-mediated effector functions by CH and LS antibodies, related to Figure 5.
(A-B) FcγR reporter activation screen of CH and LS antibodies: FcγRIIIA/CD16A (ADCC) **(A)** and FcγRIIA/CD32A (ADCP) **(B)** reporter activation induced by antibodies in the presence of cells expressing SARS-CoV-2 spike. Lucia luciferase activity is shown as fold induction relative to the isotype control. Each bar represents an antibody tested at 10 µg/mL from a single experiment. Sotrovimab, Sotro, shown for reference. **(C-D)** Dose-dependent FcγR reporter activation by LS antibodies: FcγRIIIA/CD16A (ADCC) **(C)** and FcγRIIA/CD32A (ADCP) **(D)** reporter activation shown as raw relative light units (RLU) across five-fold serial antibody dilutions starting at 10 µg/mL. Each curve represents a monoclonal antibody. Data are representative of two independent experiments performed in duplicate.

bioRxiv preprint doi: https://doi.org/10.64898/2026.09.13.750764; this version posted September 16, 2026. The copyright holder for this preprint (which was not certified by peer review) is the author/funder, who has granted bioRxiv a license to display the preprint in perpetuity. It is made available under a CC-BY 4.0 International license.

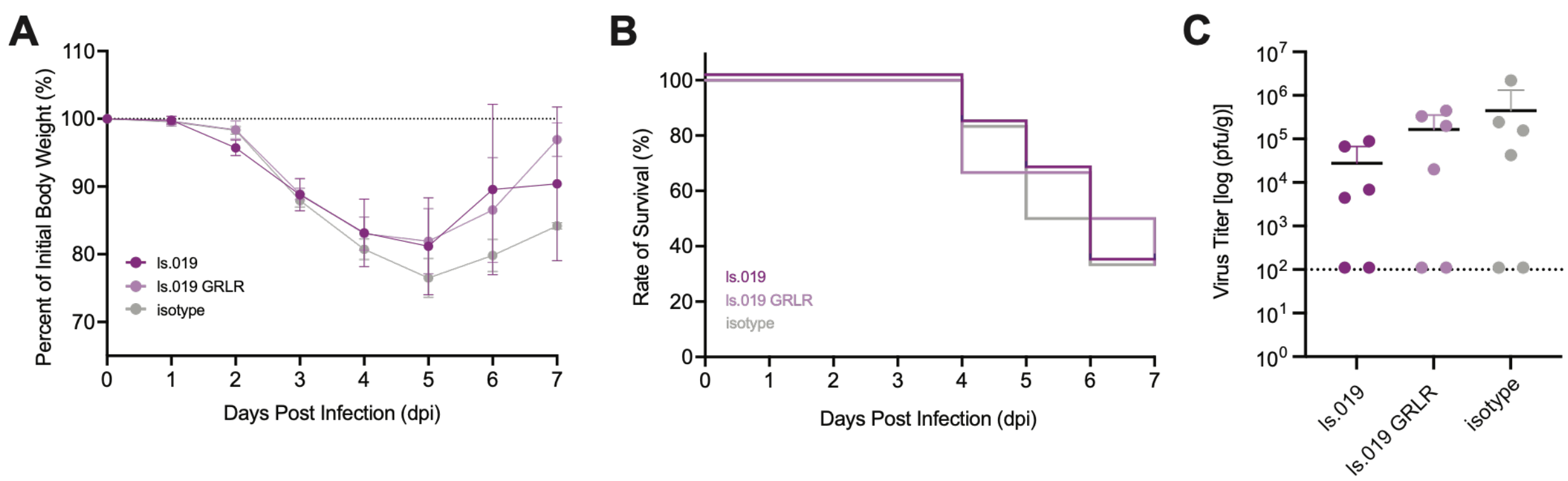

Figure S7. LS-directed antibody ls.019 does not confer protection *in vivo*, related to Figure 5.

(A) Body weight curves for BALB/c mice treated prophylactically with 500 µg of ls.019 or ls.019 GRLR compared to mice treated with isotype control antibody (500 µg), prior to i.n. inoculation with SARS-CoV-2 MA10. n = 6 per group; error bars represent the standard error of the mean but statistical analysis was not performed. **(B-C)** Corresponding survival curve **(B)** and quantification of viral titer within lungs **(C)**, related to panel (A).
